# Consumption of home-prepared meal at workplace as a predictor of glycated haemoglobin among people with type 2 diabetes in Hong Kong: a mixed-methods study

**DOI:** 10.1038/s41387-022-00188-1

**Published:** 2022-04-04

**Authors:** Heidi H. Y. Hung, Emily Ying Yang Chan, Elaine Chow, Shuk-yun Leung, Francisco Tsz Tsun Lai, Eng-kiong Yeoh

**Affiliations:** 1 The Jockey Club School of Public Health and Primary Care, The Chinese University of Hong Kong, Hong Kong SAR, China; 2 Collaborating Centre for Oxford University and CUHK for Disaster and Medical Humanitarian Response (CCOUC), The Jockey Club School of Public Health and Primary Care, The Chinese University of Hong Kong, Hong Kong SAR, China; 3 Nuffield Department of Medicine, University of Oxford, Oxford, UK; 4 François-Xavier Bagnoud Center for Health & Human Rights, Harvard University, Boston, MA USA; 5 Department of Medicine and Therapeutics, The Chinese University of Hong Kong, Hong Kong SAR, China; 6 Department of Family Medicine, New Territories East Cluster, Hospital Authority, Hong Kong SAR, China; 7 Department of Pharmacology and Pharmacy, The University of Hong Kong, Hong Kong SAR, China; 8 Laboratory of Data Discovery for Health (D24H), Hong Kong Science and Technology Park, Hong Kong SAR, China; 9 Centre for Health Systems and Policy Research, The Jockey Club School of Public Health and Primary Care, The Chinese University of Hong Kong, Hong Kong SAR, China

**Keywords:** Nutrition, Type 2 diabetes

## Abstract

**Objectives:**

There is increasing attention on association between eating patterns and diabetes control following global changes in eating patterns. There had been very limited research on the eating patterns of diabetic patients with employment, although working age population has seen the highest increase in diabetes incidence. This study aimed to identify workplace eating patterns in relation to glycaemic control among type 2 diabetic patients with employment.

**Methods:**

This is a sequential mixed-methods study. The exploratory qualitative study involved focus group interviews with 31 type 2 diabetic patients with employment, which guided the design of a subsequent cross-sectional investigation involving 185 patients with employment. Thematic analysis was conducted on the qualitative data to identify workplace eating patterns most relevant to glycaemic control. Hierarchical multiple linear regression was performed to examine association between workplace eating pattern and glycaemic control, proxied by HbA1c.

**Results:**

The focus group interviews identified frequency in the consumption of home-prepared meals (HPM) and meal hours as the major workplace eating patterns that affected glycaemic control. The cross-sectional study confirmed that regular consumption of HPM at workplace could explain variance of HbA1c, independent of socio-demographic factors, lifestyle factors and disease condition, with *R*^2^ = 0.146, *F*(14, 170) = 2.075, *p* = 0.015; adjusted *R*^2^ = 0.076. Patients who were female, in non-skilled occupation, on shift, with fixed work location and had break during work were more likely to consume HPM.

**Conclusions:**

Consumption of HPM at workplace should be promoted to facilitate better glycaemic control by type 2 diabetic patients with employment, possibly through more practical dietary advice, and workplace accommodation in terms of space and facilities. In the context of COVID-19 pandemic, consumption of HPM also meant additional protection for diabetic patients through reducing close contact exposures in restaurants.

## Introduction

Diabetes is associated with two-fold excess risk for a range of vascular diseases [1], and increases the risk of cancer death and other non-vascular death by 1.25 and 1.73 times, respectively [2]. The number of people with diabetes at working age (20–64) is expected to increase to 417 million in 2030, i.e., by about 18% in just 10 years [3]. The phenomenon of young-onset type 2 diabetes further highlighted the need to better understand the issues on diabetic control among working population, given their higher average HbA1c concentrations, earlier onset of complications [4–6] and poorer adherence to lifestyle modifications [7]. From an economic perspective, the working population affected by diabetes could lead to productivity loss in addition to healthcare cost [8–11]. From a health perspective, certain working conditions could affect the disease control of workers with diabetes, including hours worked [12, 13], shift work [14], and work-related psychosocial stress [15, 16]. Studies from this perspective were however limited and with varied quality.

Compared with a worker in the Organization for Economic Co-operation and Development (OECD) region spending on average 36 hours at work per week [17], the corresponding number for Hong Kong, being an economically advanced Asian city, is at 44 [18]. For an employed person, the work environment shapes heavily her daily routine, including eating patterns. Due to the changing eating patterns across the world, e.g., more frequent eating-out, irregular meal times, there has been increasing attention on the association between eating patterns and the risk and control of diabetes and cardiovascular diseases [19]. Some eating patterns in particular, including breakfast skipping, lower meal frequency, late-night dinner, have been found to be associated with glycaemic control among people with diabetes [20, 21]. None of these studies however focused on the situation of diabetic patients with employment and eating patterns conditioned by the workplace. Given the potential impact that eating patterns at workplace may have on glycaemic control and the impact of working conditions on eating patterns, this study aimed to identify major workplace eating patterns in relation to glycaemic control among diabetic patients with employment.

## Methods

### Conceptual framework

**Fig. 1:**
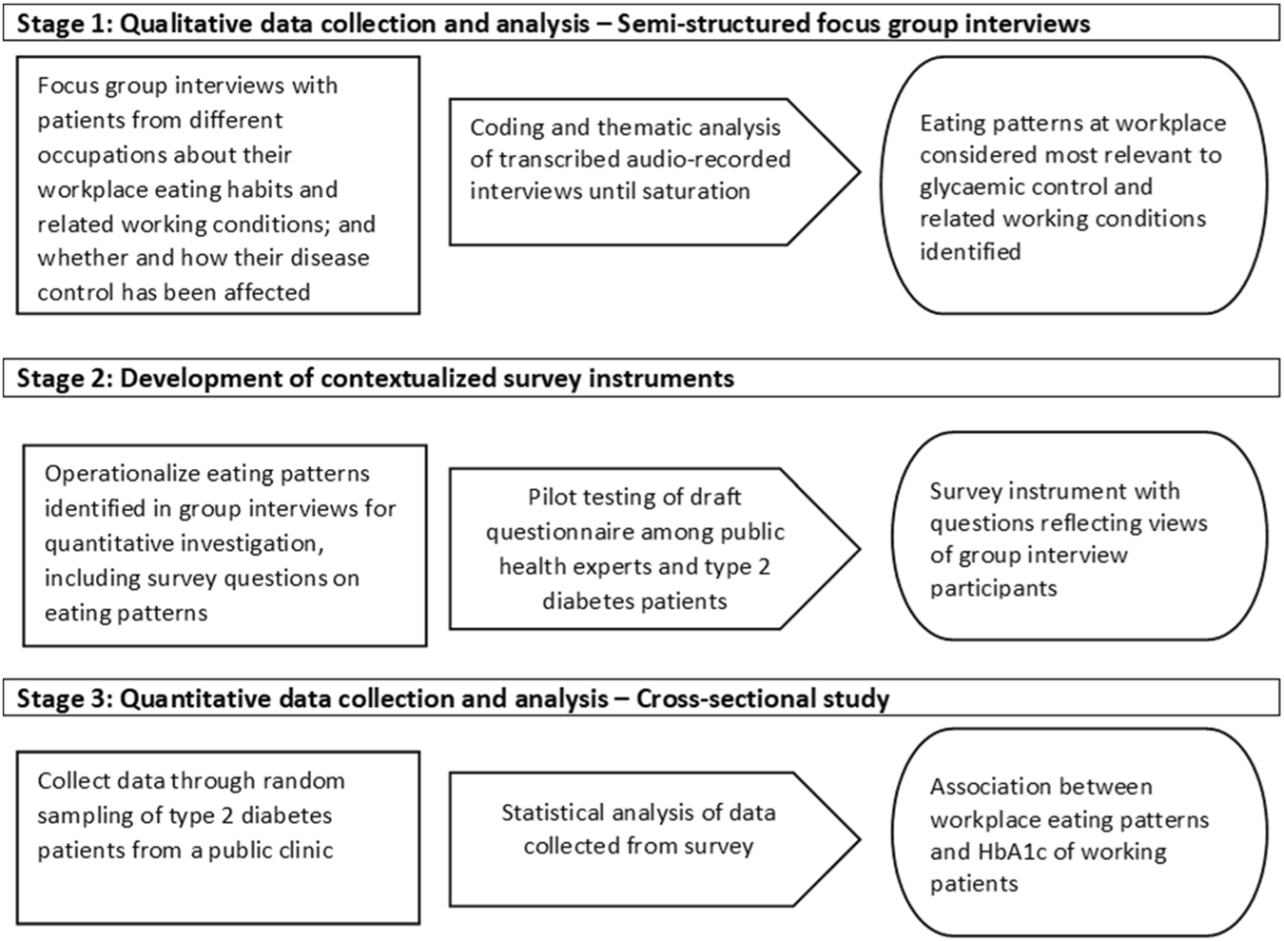
An overview of the study design: exploratory sequential mixed-methods study. Focus groups were first conducted to identify workplace eating habits and related working conditions that were relevant to glycaemic control (stage 1); findings from focus groups guided the development of the survey instrument of the subsequent cross-sectional study (stage 2); and finally statistical analysis was conducted to confirm possible association between workplace eating habits and glycaemic control (stage 3).

This was a sequential mixed-methods study that consisted of an exploratory qualitative study (semi-structured focus group interviews) and a subsequent cross-sectional study, with independent sampling (Fig. 1). Mixed methods study was employed because the relationship between work environment and diabetes control was a multidimensional issue that had not been well researched on [22]. Findings from the qualitative study guided the operationalization of workplace eating patterns in the subsequent quantitative stages of the investigation [23].

### Data source and subjects

For both the qualitative and quantitative parts of the study, eligible participants were those aged between 18 and 65, with type 2 diabetes diagnosed for at least 6 months, and had been in the same employment for at least 6 months before the date of interview/ survey.

The semi-structured focus group interviews were conducted from April to July 2019 to identify eating patterns at the workplace that the patients considered most relevant for glycaemic control, and the working conditions that affected workplace eating patterns. Participants were recruited from the diabetes complication screening sessions of the Prince of Wales Hospital (PWH), one of the largest public hospitals in Hong Kong. Heterogenous purposive sampling was used to ensure diversity in the participants’ occupations, diabetes regimens and age-gender mix (Appendix 1). As the discussion topic generally did not involve personal issues, and interaction in experience sharing among patients would be valuable, focus group was considered an appropriate methodology [24]. To develop rapport, the focus group moderator had an informal discussion with individual participant to explain the study and to understand their work environment; and they signed a standardized consent form before the focus groups began. The focus groups lasted between 50 and 75 min. All focus groups were moderated by the same investigator (the first author HHYH) with a discussion guide to minimize interviewer bias. The discussion guide was developed based on literature review and preliminary discussion with diabetes specialist and union representatives, and was reviewed by public health researchers. The focus groups were conducted face-to-face without presence of non-participants, and the discussion was audio-recorded with verbal consent of the participants. The audio records were then transcribed verbatim and anonymized. Field notes were taken by the moderator during the focus group interviews to assess data saturation. Participants were recruited until saturation was reached with no new preliminary themes identified.

Findings from the qualitative study and literature review informed the design of the cross-sectional study, in particular the development of the survey instrument. The draft questionnaire was reviewed by six non-patient individuals with knowledge in public health/medicine; and fluency in Cantonese and Chinese characters. The revised questionnaire was then piloted among 28 type 2 diabetic patients with employment, who were invited to comment on the questionnaire, in particular its clarity, relevance and length.

Participants of the cross-sectional study were recruited from the Fanling Family Medicine Center (FLFMC), which was one of the largest public clinics in the same organizational and geographical cluster as PWH, providing regular follow-up appointments for diabetic patients. Participants were randomly selected from the appointment list of the FLFMC for January 2020, and were approached and invited to participate in the study when they attended the appointments. Every participant signed a standardized consent form before filling out the self-administered questionnaire. Trained researchers were on site to obtain consent, explain the study and clarify questions that the participants had, so as to address information bias to some extent. Primary data collected included participants’ demographics, socioeconomic status, employment situation, disease conditions and regimens, and eating patterns at workplace. Electronic records of the participants’ latest HbA1c levels (%) available three months after the completion of the questionnaire were retrieved from FLFMC database.

HbA1c was chosen to proxy glycaemic control in this study as it had been recognized as the primary diabetes outcome of metabolic control, given its association with diabetic complication [25], and mortality (all-cause and cardiovascular) [26]. Dependent variable was the actual HbA1c level (%) of patients. Independent variables included the eating patterns identified in focus group interviews, i.e., consumption of home-prepared meals at workplace (regular/occasional/never) and meal hours (regular/irregular). Confounders included socio-demographic factors of age, sex, education attainment (primary and below/secondary/tertiary), personal monthly income (HK$0–9999/HK$10,000–29,999/HK$30,000, and above) and occupation (non-skilled/medium-skilled/highly-skilled); lifestyle factors of smoking habit (current smoker or not) and performance of exercise outside home and work per week (yes/no); and disease conditions of diabetes duration (years) and presence of comorbidities (yes/no). The sample size for the cross-sectional study was calculated by G*Power 3.1.9.4, based on the assumptions of a small effect size [27], confidence interval of 95%, standard deviation of 0.5, a power of 80%, and with 12 independent variables for multiple linear regression [28].

Ethical approvals for both the qualitative study and cross-sectional study were granted by The Joint Chinese University of Hong Kong—New Territories East Cluster Clinical Research Ethics Committee (The Joint CUHK-NTEC CREC) which covered the sites where patients were recruited. The Strengthening the Reporting of Observational Studies in Epidemiology Statement (STROBE) [29] for reporting cross-sectional studies and the Consolidated Criteria for Reporting Qualitative Research (COREQ) checklists [30] were followed in the reporting of this study (Appendix 2).

### Data analysis

Inductive thematic analysis under essentialist approach at semantic level was conducted for the focus group interviews [31]. Guidelines by Braun and Clarke were adopted in conducting the thematic analysis [32]. Transcript from each group was first reviewed in detail to familiarize with the data and search for patterns with notes on margin. Initial coding of the transcribed discussion records was then done, followed by collating of codes into potential themes, reviewing of themes for internal homogeneity and external heterogeneity [33], and defining of final themes. The data analysis process was managed by NVivo 12.

For the cross-sectional study, demographics, socioeconomic status, disease conditions, regimens and working conditions of participants were summarized in descriptive statistics. Linear-by-linear association tests and analysis of variance (ANOVA) tests were conducted to profile diabetic patients, who displayed the workplace eating pattern considered most critical to glycaemic control in focus group interviews. Correlations between HbA1c and continuous covariates: age, disease duration, and working hours were assessed by Spearman’s rank-order correlation coefficient. Group comparison of HbA1c according to socioeconomic status and workplace eating patterns with related working conditions was conducted by using Kruskal–Wallis *H*-test or Mann–Whitney *U*-test. Hierarchical multiple linear regression was then conducted to identify workplace eating patterns that were predictive of HbA1c level, after adjustments of potential confounders. Planned sensitivity analysis was conducted by removing insulin-treated patients given their extreme disease severity, and post-hoc sensitivity analysis removing outliers in the outcome variable was also performed. There was no missing data for the variables included in the multiple regression analyses. All significance tests were two-tailed and a *P* value < 0.05 was considered statistically significant. Data from the cross-sectional study were analysed using IBM Statistical Package for the Social Sciences (SPSS) version 22.

## Results

### Focus groups: participants

13 out of the 50 participants recruited withdrew from the focus group interviews citing clash with work commitments; and another six were not able to have their views included in the study as the scheduled focus groups could not proceed due to insufficient attendance. Eventually, nine focus groups involving 31 participants were covered in this study. As most of the focus groups were conducted right after the screening sessions to accommodate the schedule of working patients, group size tended to be small, with a minimum of three [34]. The median age was 54 (range 26–63), 14 (45%) were female, the average disease duration was 10 years and 18 (58%) were insulin-treated. A wide range of occupations were covered, with 16% from non-skilled occupation groups, 49% from medium-skilled and 29% from highly-skilled. The participants’ characteristics largely reflected our heterogeneous sampling plan (Appendix 1); although insulin-treated patients had been somewhat over-represented and patients from highly-skilled occupation groups had been under-represented (Table 1).

**Table 1:** Characteristics of participants in focus group interviews (*n* = 31).

Focus group	Participant number	Age	Sex	Occupation	Disease duration	Regimen	
						Diabetic drugs	Insulin
F1	1	42	M	Security guard	16 m	√	√
F1	2	55	M	Retail salesperson	16 y	√	√
F1	3	50	F	Hospital chemist	10 y	√	
F1	4	43	M	Bank manager	10 m	√	
F2	1	55	M	Project assistant	16 y	√	√
F2	2	49	F	Telephone operator	20 y	√	√
F2	3	62	M	Hotel manager	7 y	√	√
F3	1	54	F	Clerical	17 y	√	√
F3	2	58	F	Clerical	4 y	√	
F3	3	49	F	Office assistant	9 y	√	
F3	4	63	F	Clerical	5 y	√	
F4	1	58	M	Civil engineer	1 y	√	
F4	2	60	M	Merchandizer	25 y	√	√
F4	3	53	M	Goods delivery	11 y	√	√
F5	1	26	F	Travel agent (ticketing)	6 m	√	
F5	2	29	F	Retail cashier	2 y	√	√
F5	3	53	M	Driver (cross-border truck)	5 y	√	
F5	4	49	M	Driver (delivery truck)	10 y	√	√
F6	1	55	M	Driver (taxi)	10 y	√	√
F6	2	59	M	Construction painter	10 y	√	√
F6	3	54	F	Manager	9 y	√	√
F7	1	58	F	Nurse	10 y	√	
F7	2	52	M	Renovation company owner	12 y	√	
F7	3	51	F	Retail cashier	7 m		
F7	4	63	M	Construction site laborer	10 y	√	√
F8	1	58	M	Engineer	20 y	√	√
F8	2	63	F	Dishwasher	6 y	√	
F8	3	46	F	Waitress	28 y	√	
F9	1	39	M	Retail salesperson	NA	√	√
F9	2	57	M	Company owner	20 y	√	√
F9	3	42	F	Waitress	1 y	√	

### Focus groups: key themes

Five themes had been identified from the focus group discussion over working conditions, eating patterns and glycaemic control.

First, eating out and eating at irregular time were considered as the workplace eating patterns that were most detrimental to glycaemic control. There was a clear consensus that eating out was a major barrier to healthy diet, and consumption of home-prepared meal (HPM) was beneficial for glycaemic control. Some participants considered it possible to reduce the frequency of eating out, although significant challenges were cited. Eating at irregular time on the other hand was a non-modifiable situation for the relevant patients since it was due to their job nature, e.g., when lunch hours depended on the volume of business/workload of a particular day or flow of traffic.

Secondly, there was a strong sense of powerlessness among patients eating out, as they felt they had little control over the composition of restaurant meals, in particular the level of oil, sugar and salt used, the sugary drinks and minimal amount of vegetables that came in set meals. Certain coping strategies were adopted by some patients, including asking for a smaller portion, opting for food that was prepared with healthier cooking method (e.g., steaming), avoiding food with lots of sauces. Most of the patients however agreed that such strategies were not sufficient to ensure that their meals were diabetes-friendly.

Thirdly, participants who consumed HPM found it possible to have diets that were beneficial to glycaemic control. They felt that they were able to control what they ate and showed a reasonable level of confidence in adhering to healthy diet. Patients who consumed HPM however were in the minority: 4 out of the 31 participants, and they were all female.

Fourthly, eating out was the only feasible meal option for some patients due to some non-modifiable work location settings: no fixed work location, and/or no suitable facilities. The absence of fixed work location meant that patients had nowhere to store and consume HPM. A notable example was drivers. Patients who were professional drivers in the study reported that they could only visit whatever eateries that were near their location on a particular day, coupling with irregular meal hours for many.

Finally, there was strong sentiment that the dietary advice received from dieticians in the hospital/clinic were impractical and difficult to follow. For patients with work setting possible for HPM but constantly ate out, many indicated that they did not consider HPM because it was impossible to adhere to the dieticians’ advice in meal preparation. They expressed a strong desire for the dietary advice to take into account the actual life situation of working patients (e.g., energy requirement at work). Unlike patients with no fixed work location, a good number of patients might have the potential to switch to HPM, if the difficulties they saw in following dieticians’ advice could be ameliorated to some extent.

**Fig. 2:**
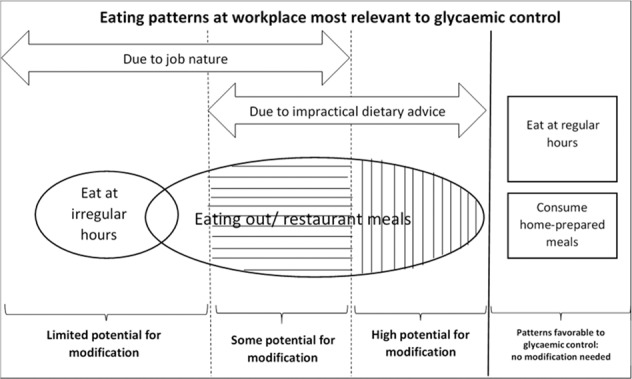
Thematic map of focus group interviews analysis. Eating out was found to be the most common and relevant workplace eating pattern that hindered glycaemic control among the focus group participants. Those who ate out due to job nature had little potential for changing this pattern, while those who ate out due to impractical dietary advice might have the highest potential for modification.

Table 2 summarized the details of each of the above theme with quotes from participants. In summary, eating out in restaurants/consuming HPM and having meals at irregular/regular hours were the two workplace eating patterns identified as most relevant to glycaemic control at the focus group interviews. Many patients found the advice they obtained from dieticians impractical, creating a barrier to prepare HPM, coupling with some unfavorable working conditions for some, such as long working hours. For some patients, consuming HPM was not feasible primarily because of their work location settings, which were not non-modifiable. Similarly, eating at irregular hours was also considered as inevitable due to job nature. A broader understanding over the relationship between the themes was presented in a thematic map (Fig. 2). Findings from the focus group interviews were fed into the design of the cross-sectional study.

**Table 2:** Summary of themes identified from focus group discussion over working conditions, eating patterns and glycaemic control (*n* = 31).

	Themes	Details	Quotes
1.	The what and the when: Eating out and eating at irregular time as the workplace eating patterns considered most detrimental to glycaemic control	• Eating out: a major barrier to healthy diet, and consumption of home-prepared meal was beneficial for glycaemic control, some due to non-modifiable work conditions while some considered change possible • Irregular hours: non-modifiable work condition, overeat/snacking, hypo	“If I know it will be a busy day with lots of goods to deliver, I would rather have a very big breakfast, skip my lunch then have an early dinner at around 5 pm. It sure does not work well with my medication schedule.” (F5-4, delivery truck driver)
2.	The powerlessness with eating out	• Little control over the composition of restaurant meals, in particular the level of oil, sugar and salt used • Sugary drinks and minimal amount of vegetables • Some coping strategies but insufficient	“Even if you ask for “3-low” [low sugar, low fat, low sodium], they may not be able to do it for you in the restaurants. Many restaurants do not serve vegetables in their lunch set, so I usually eat more vegetables when I have dinner at home” (F6-3, manager)
3.	Those consuming home-prepared meal (HPM) felt confident and in control	• Participants who consumed HPM found it possible to have diets that were beneficial to glycaemic control • Not a common practice	“I find it much easier to have a healthy diet by bringing in my own homemade lunch. I have a lunch box with three compartments, and it helps me to be clear with how much of what I am eating.” (F5-1, travel agent ticketing officer)
4.	Eating out was inevitable for some primarily because of work location settings	• For some patients, eating out was the only meal option due to some non-modifiable work location settings: no fixed work location, and/ or no suitable facilities	“I eat out every day for lunch and just anywhere near the construction site. We have no table, no chair, no fridge, no microwave, nothing! How do we bring in home-prepared lunch?” (F7-4, construction site laborer)
5.	HPM could be facilitated by more practical dietary advice	• Some patients indicated that they did not consider HPM because it was impossible to adhere to the dieticians’ advice in meal preparation • Strong desire for the dietary advice to take into account the actual life situation of working patients	“What the dieticians said was completely impractical. You could follow only if you don’ have to work. They don’t put themselves in our shoes and only talk about ideals. “ (F9-1, salesperson)

### Cross-sectional study: participants

In total, 422 type 2 diabetic patients were approached, with 185 patients with employment completed the questionnaire (Supplementary Fig. 1 in Appendix 3). The characteristics of the study participants were shown in Table 3, stratified by the eating pattern of consuming HPM during work hours. Among 185 participants, 73.5% were male, with the mean (SD) age of 56.7 (6.0) and mean (SD) diabetes duration of 6.7 (5.7) years. 21.1% of all participants consumed HPM regularly during work hours and 67.6% consumed only restaurant meals, with the rest having HPM occasionally. Consumption of HPM during work hours was associated with gender, occupation, diabetes regimen, and certain working conditions: patients who were female (*p* = 0.03), in non-skilled occupation (*p* = 0.002), taking no diabetic medication (*p* = 0.032), on shift (*p* = 0.046), with fixed work location (*p* < 0.001) and had break during work (*p* = 0.004) were more likely to consume HPM.

**Table 3:** Characteristics of participants in cross-sectional study (*n* = 185).

	Regular HPM#		Occasional HPM#		Restaurant meal only		Total		*p*-trend^‡^
No. of subjects	39	(21.1)	21	(11.4)	125	(67.6)	185		
Age (years)^†^	57.2	(4.9)	58.3	(4.5)	56.2	(6.5)	56.7	(6.0)	0.190
Male	22	(56.4)	14	(66.7)	100	(80.0)	136	(73.5)	0.003
Married	35	(89.7)	16	(76.2)	103	(82.4)	154	(83.2)	0.389
Born in Hong Kong	23	(59.0)	13	(61.9)	87	(69.6)	123	(66.5)	0.196
*Education*									0.944
Primary and below	6	(15.4)	6	(28.6)	20	(16.0)	32	(17.3)	
Secondary	25	(64.1)	12	(57.1)	83	(66.4)	120	(64.9)	
Tertiary	8	(20.5)	3	(14.3)	22	(17.6)	33	(17.8)	
*Income level*									0.067
$0–9999	12	(30.8)	8	(38.1)	27	(21.6)	47	(25.4)	
$10,000–29,999	23	(59.0)	12	(57.1)	75	(60.0)	110	(59.5)	
$30,000 or above	4	(10.3)	1	(4.8)	23	(18.4)	28	(15.1)	
*Occupation*									0.002
Non-skilled	16	(41.0)	7	(33.3)	18	(14.4)	41	(22.2)	
Medium-skilled	15	(38.5)	10	(47.6)	69	(55.2)	94	(50.8)	
Highly-skilled	8	(20.5)	4	(19.0)	38	(30.4)	50	(27.0)	
Diabetes duration (years)^†^	6.2	(5.9)	7.8	(6.1)	6.7	(5.4)	6.7	(5.7)	0.743
HbA1c (%)^†^	6.62	(0.51)	7.16	(0.97)	7.08	(0.92)	6.99	(0.88)	0.010
*Diabetes regimens*									0.032
No medication	7	(17.9)	1	(4.8)	5	(4.0)	13	(7.0)	
Diabetes drugs only	31	(79.5)	19	(90.5)	118	(94.4)	168	(90.8)	
Insulin	1	(2.6)	1	(4.8)	2	(1.6)	4	(2.2)	
Presence of comorbidities	32	(82.1)	17	(81.0)	95	(76.0)	144	(77.8)	0.396
Current smoker	6	12.0	4	4.3	34	82.9	44	23.8	0.112
At least one exercise/week	24	(61.5)	11	(52.4)	65	(52.0)	100	(54.1)	0.342
Shift work	10	(25.6)	1	(4.8)	14	(11.2)	25	(13.5)	0.046
Working hours^†^^a^	46.7	15.4	54.8	16.1	48.9	12.8	49.1	13.9	0.104
Fixed work location	38	(97.4)	12	(57.1)	80	(64.0)	130	(70.3)	<0.001
Break during work	34	(87.2)	19	(90.5)	83	(66.4)	136	(73.5)	0.004
Regular meal hours	33	(84.6)	15	(71.4)	91	(72.8)	139	(75.1)	0.170

*HPM* home-prepared meal, data are *N* (%) unless specified otherwise.

^†^Mean (SD).

^‡^Differences between occupation groups assessed by Linear-by-Linear Association test or ANOVA test.

^a^*n* = 178 due to missing data.

### Cross-sectional study: workplace eating patterns associated with HbA1c

A Kruskal–Wallis *H*-test indicated that there was a statistically significant difference between HbA1c of patients consuming HPM regularly and those consuming only restaurant meals (H(2) = 9.579, *p* = 0.008). In addition, there was a low, positive correlation between diabetes duration and HbA1c, which was statistically significant (*r*_s_ = 0.299, *p* < 0.001) (Table 4). There was no group difference in HbA1c for the other eating pattern identified, e.g., eating at regular/irregular hours, nor for the related working conditions.

**Table 4:** Correlation between independent variables and HbA1c level/Comparison of HbA1c levels between groups categorized by different independent variables (*n* = 185).

	Test statistics	*p*-value
*Sociodemographic factors*		
Age (years)	*r*_s_ = −0.069	0.353
Sex	U = 3212.0	0.709
Education (tertiary/secondary/primary)	*H*(2) = 2.320	0.314
Income level (high/middle/low)	*H*(2) = 0.290	0.865
Occupation (highly skilled/medium-skilled/non-skilled)	*H*(2) = 3.799	0.150
*Lifestyle factors*		
Cigarette consumption (smoker/non-smoker)	*U* = 3009.5	*0.765*
Exercise outside home and work (yes/no)	*U* = 3924.0	*0.369*
*Disease condition*		
Prescence of comorbidities	*U* = 2705.0	0.414
Diabetes duration (years)	*r*_s_ = 0.299	<0.001
*Workplace eating patterns and related working conditions*		
Home-prepared meal (regular/occasional/never)	*H*(2) = 9.579	0.008
Meal hours (regular/irregular)	*U* = 2942.0	0.418
Work schedule (shift/non-shift)	*U* = 1896.0	0.676
Work location (fixed/mobile)	*U* = 3513.5	0.853
Break at work (break/no break)	*U* = 2876.0	0.156
Working hours^†^	*r*_s_ = 0.033	0.662

^†^*n* = 178 due to missing data

### Hierarchical multiple linear regression—workplace eating patterns predictive of HbA1c

A hierarchical multiple linear regression (enter method) was performed to determine if consumption of HPM at workplace was predictive of HbA1c, after adjusting for socio-demographic factors, disease condition and lifestyle factors. See Table 5 for full details on each regression model. Model 1 and Model 2, i.e., socio-demographic and lifestyle factors, were not able to explain the variance in HbA1c. Model 3, with the addition of disease duration to the prediction of HbA1c, led to a statistically significant increase in *R*^2^ of 0.055, *F*(2, 172) = 5.293, *p* = 0.006. Model 4, with the addition of consumption of HPM at workplace to the prediction of HbA1c, also led to a statistically significant increase in *R*^2^ of 0.044, *F*(2, 170) = 4.339, *p* = 0.015. The full model (Model 4) was statistically significant, *R*^2^ = 0.146, *F*(14, 170) = 2.075, *p* = 0.015; adjusted *R*^2^ = 0.076. Our results confirmed that regular consumption of HPM at workplace could explain variance of HbA1c, independent of socio-demographic factors, lifestyle factors and disease condition.

**Table 5:** Hierarchical multiple linear regression models predicting HbA1c (*n* = 185).

	HbA1c (%)							
	Model 1		Model 2		Model 3		Model 4	
Variable	*B*	*β*	*B*	*β*	*B*	*β*	*B*	*β*
Age	−0.069	−0.035	−0.085	−0.043	−0.055	−0.028	0.026	0.013
Sex	−0.004	−0.027	−0.003	−0.022	−0.009	−0.063	−0.006	−0.039
Education level (primary)	−0.021	−0.009	−0.058	−0.025	−0.032	−0.014	−0.132	−0.057
Education level (secondary)	−0.204	−0.111	−0.223	−0.122	−0.226	−0.123	−0.288	−0.157
Occupation (non-skilled)	−0.257	−0.122	−0.251	−0.119	−0.216	−0.103	−0.105	−0.050
Occupation (medium-skilled)	−0.342	−0.195	−0.353	−0.202	−0.311	−0.178	−0.286	−0.163
Monthly income (HK$0–9999)	0.267	0.133	0.280	0.140	0.283	0.141	0.281	0.140
Monthly income (HK$10,000–29,999)	0.343	0.193	0.369	0.207	0.329	0.185	0.337	0.189
Current smoker			0.003	0.002	0.003	0.002	−0.004	−0.002
Exercise outside home and work			−0.152	−0.087	−0.131	−0.074	−0.107	−0.061
Diabetes duration					0.037*	0.239	0.035*	0.226
Presence of comorbidities					0.120	0.057	0.103	0.049
Consumption of HPM (regular)							−0.475*	−0.221
Consumption of HPM (occasional)							0.002	0.001
*R*^2^	0.040		0.047		0.102		0.146	
Δ*R*^2^	0.040		0.007		0.055*		0.044*	

*HPM* home-prepared meal.

*N* = 185. *p < 0.05.

Appendix 4 demonstrated that our cross-sectional data met the various assumptions for multiple linear regression, including tests of linearity, homoscedasticity, multicollinearity, normality of residues and outliers. It should be note that the three potential outliers (defined as those with studentized deleted residuals greater than ±3 standard deviations) were not removed from the main analysis since they were neither high leverage points nor influential points, and it was unlikely that the outliers existed due to input error as they were retrieved directly from electronic records of the clinic.

Sensitivity analyses were conducted by (a) removing insulin-treated patients given their extreme disease severity (*n* = 181) and (b) removing the outliers in the outcome variable described above (*n* = 182). The results were largely similar to those from the main analysis (Supplementary Table 1 in Appendix 5), indicating robustness of the primary results.

## Discussion

Our analysis provided evidence that regular consumption of HPM during work hours was associated with lower HbA1c level for patients with type 2 diabetes, after adjusting for socio-demographic factors, lifestyle factors and disease condition. It had been found that consumption of HPM during work hours was not a common practice, with female, non-skilled workers and those with fixed work location, on shift and have break during work more likely to do so. These working conditions however were not associated with HbA1c. The absence of fixed work location and impractical dietary advice were possible barriers to consumption of HPM during working hours. Our study was the first to examine workplace eating patterns in relation to HbA1c, thus shedding light on how type 2 diabetic patients with employment could improve their disease control. In the context of the COVID-19 pandemic, it might be the case that consumption of HPM would bring “double health benefits” for diabetic patients with employment: better glycaemic control, and protection from COVID-19 through reducing close contact exposures at restaurants [35].

Eating out had been on an upward trend globally, and was associated with less healthy diet, including higher energy, higher fat and lower micronutrient intake [36], increased diabetes risk [37], and insulin resistance [38]. On the other hand, frequent consumption of HPM was found to be associated with lower risk of developing type 2 diabetes [39]. The impact of eating out on glycaemic control of people with diabetes has however received little attention; and with eating out being more common among working population [40], the eating pattern of diabetic patients with employment merited particular attention. Findings from our study supported the promotion of HPM at workplace for better glycaemic control, and provided some evidence that more practical dietary advice and accommodation at work settings might encourage consumption of HPM at workplace. Measures should be taken to provide dietary advice, taking into account the schedules and needs of working patients, e.g., easy-to-follow cooking instructions and recipe, more discussion on the energy needs of working patients with dieticians. In terms of workplace accommodation, companies should be encouraged to facilitate employees with diabetes to bring HPM, by providing space and facilities for storage, processing and consumption. For patients who could not switch to HPM due to their working conditions, restaurant-based interventions should be considered, e.g., menu labeling [41], increased healthy food choices [42].

The main strength of our investigation lied in its study design, as the qualitative analysis enabled the quantitative study to focus on questions relevant to the real-life situation of employed diabetic patients, and the quantitative analysis confirmed some of the findings from the qualitative study. Sampling from major public hospital and clinic meant that our participants represented a wide range of socio-economic background. Unlike other studies on socioeconomic factors and glycaemic control, which were often dominated by retired patients [43, 44], this study focused specifically on working patients. Our study however was also subject to a few limitations. We had no information on the nutritional content, ingredients, and cooking methods used in the HPM. However, it was clear from our qualitative study that patients considered their diets much healthier when they ate at home, and this was in line with a previous study which found an association between cooking dinner at home and consumption of healthier diet [45]. Second, confounding effects on HbA1c level from factors not covered in our study could not be ruled out, e.g., genetic determinants [46]. Third, as all predictor variables were self-reported, there could be some measurement errors. Fourth, the sample size for the cross-sectional study was relatively small, especially for the number of patients consuming HPM, which limited the generalizability of the study. Fifth, it had been noted that while participants who did not consume HPM regularly had higher mean HbA1c, a higher percentage of them were already on diabetic drugs at the same time, as compared with those who consumed HPM regularly. Our study was not able to verify if it had any implications on the effectiveness of the regimen. Finally, the observational nature of the study meant causal relationship between HPM at workplace and HbA1c could not be established. Our findings nonetheless pointed to the potential of larger scale cohort studies in future, on the beneficial effect of consumption of HPM during working hours for people with type 2 diabetes.

## Supplementary information

Supplementary Materials

## Data Availability

The data that support the findings of this study are not openly available for protection of participants’ privacy over medical data; and are available from the corresponding author upon reasonable request.

## References

[1] The Emerging Risk Factors Collaboration. (2010). Diabetes mellitus, fasting blood glucose concentration, and risk of vascular disease: a collaborative meta-analysis of 102 prospective studies. Lancet.

[2] Emerging Risk Factors Collaboration. Diabetes mellitus, fasting glucose, and risk of cause-specific death. N Engl J Med. 2011;364:829–41. 10.1056/NEJMoa1008862 PMC4109980 21366474

[3] International Diabetes Federation. IDF Diabetes Atlas, 9th edn. 2019. https://www.diabetesatlas.org.

[4] Song SH.Hardisty CA, Early onset type 2 diabetes mellitus: a harbinger for complications in later years-clinical observation from a secondary care cohort. QJM. 2009;102:799–806. http://www.ncbi.nlm.nih.gov/pubmed/19734298 10.1093/qjmed/hcp121 19734298

[5] Al-Saeed AH, Constantino MI, Molyneaux L, D’Souza M, Limacher-Gisler F, Luo C.et al. An inverse relationship between age of type 2 diabetes onset and complication risk and mortality: the Impact of Youth-Onset Type 2 Diabetes. Diabetes Care. 2016;39:823–9. http://care.diabetesjournals.org/lookup/doi/10.2337/dc15-0991 10.2337/dc15-0991 27006511

[6] Chan JCN, Lau ESH, Luk AOY, Cheung KKT, Kong APS.Yu LWL,et al. Premature mortality and comorbiditie s in young-onset diabetes: a 7-year prospective analysis. Am J Med. 2014;127:616–24. https://linkinghub.elsevier.com/retrieve/pii/S0002934314002721 10.1016/j.amjmed.2014.03.018 24680795

[7] Yeung RO, Zhang Y, Luk A, Yang W, Sobrepena L.Yoon K-H,et al. Metabolic profiles and treatment gaps in young-onset type 2 diabetes in Asia (the JADE programme): a cross-sectional study of a prospective cohort. Lancet Diabetes Endocrinol. 2014;2:935–43. https://linkinghub.elsevier.com/retrieve/pii/S2213858714701378 10.1016/S2213-8587(14)70137-8 25081582

[8] Kirigia JM, Sambo HB, Sambo LG, Barry SP. Economic burden of diabetes mellitus in the WHO African region. RBMC Int Health Hum Rights. 2009;9:6. https://bmcinthealthhumrights.biomedcentral.com/articles/10.1186/1472-698X-9-6 10.1186/1472-698X-9-6 PMC2674592 19335903

[9] Bommer C, Sagalova V, Heesemann E, Manne-Goehler J, Atun R, Bärnighausen T.et al. Global economic burden of diabetes in adults: projections from 2015 to 2030. Diabetes Care. 2018;41:963–70. http://care.diabetesjournals.org/lookup/doi/10.2337/dc17-1962 10.2337/dc17-1962 29475843

[10] Economic Costs of Diabetes in the U.S. in 2017. Diabetes Care. 2018;41:917–28. http://care.diabetesjournals.org/lookup/doi/10.2337/dci18-0007 10.2337/dci18-0007 PMC5911784 29567642

[11] Png ME, Yoong J, Phan TP, Wee HL. Current and future economic burden of diabetes among working-age adults in Asia: conservative estimates for Singapore from 2010–2050. BMC Public Health. 2016;16:153. http://www.biomedcentral.com/1471-2458/16/153 10.1186/s12889-016-2827-1 PMC4754926 26880337

[12] Wendel CS, Shah JH, Duckworth WC, Hoffman RM, Mohler MJ, Murata GH. Racial and ethnic disparities in the control of cardiovascular disease risk factors in Southwest American veterans with type 2 diabetes: the Diabetes Outcomes in Veterans Study. BMC Health Serv Res. 2006;6:58. 10.1186/1472-6963-6-58 PMC1513224 16716235

[13] Walker RJ, Gebregziabher M, Martin-Harris B, Egede LE. Relationship between social determinants of health and processes and outcomes in adults with type 2 diabetes: validation of a conceptual framework. BMC Endocr Disord [Internet]. 2014;14:82. http://www.ncbi.nlm.nih.gov/pubmed/25298071 10.1186/1472-6823-14-82 PMC4203970 25298071

[14] Knutsson A, Kempe A. Shift work and diabetes-a systematic review. Chronobiol Int [Internet]. 2014;31:1146–51. http://www.ncbi.nlm.nih.gov/pubmed/25290038 10.3109/07420528.2014.957308 25290038

[15] Trief PM, Aquilino C, Paradies K, Weinstock RS. Impact of the work environment on gl ycemic control and adaptation to diabetes. Diabetes Care. 1999;22:569–74. http://care.diabetesjournals.org/cgi/doi/10.2337/diacare.22.4.569 10.2337/diacare.22.4.569 10189533

[16] Annor FB, Roblin DW, Okosun IS, Goodman M. Work-related psychosocial stress and glycemic control among working adults with diabetes mellitus. Diabetes Metab Syndr Clin Res Rev. 2015;9:85–90. https://linkinghub.elsevier.com/retrieve/pii/S1871402115000168 10.1016/j.dsx.2015.02.003 25818923

[17] Organisation for Economic Co-operation and Development (OECD). Average usual weekly hours worked on the main job. 2017. https://stats.oecd.org/index.aspx?DataSetCode=ANHRS#

[18] Hong Kong Census and Statistics Department. Median hours of work during the 7 days before enumeration of employed persons by sex and industry of main employment. 2017. https://www.censtatd.gov.hk/hkstat/sub/sp200.jsp?tableID=016&ID=0&productType=8

[19] St-Onge M-P, Ard J, Baskin ML, Chiuve SE, Johnson HM, Kris-Etherton P, et al. Meal timing and frequency: implications for cardiovascular disease prevention: a Scientific Statement From the American Heart Association. Circulation. 2017. https://www.ahajournals.org/doi/10.1161/CIR.0000000000000476 10.1161/CIR.0000000000000476 PMC8532518 28137935

[20] Reutrakul S, Hood MM, Crowley SJ, Morgan MK, Teodori M, Knutson KL. The relationship between breakfast skipping, chronotype, and glycemic control in type 2 diabetes. Chronobiol Int. 2014;31:64–71. http://www.tandfonline.com/doi/full/10.3109/07420528.2013.821614 10.3109/07420528.2013.821614 24094031

[21] Ahola AJ, Mutter S, Forsblom C, Harjutsalo V, Groop P-H. Meal timing, meal frequency, and breakfast skipping in adult individuals with type 1 diabetes—associations with glycaemic control. Sci Rep. 2019;9:20063. http://www.nature.com/articles/s41598-019-56541-5 10.1038/s41598-019-56541-5 PMC6934661 31882789

[22] Curry L, Nunez-Smith M (2014). Mixed methods in health sciences research: a practical primer.

[23] O’Cathain A, Murphy E.Nicholl J, Why, and how, mixed methods research is undertaken in health services research in England: a mixed methods study. BMC Health Serv Res. 2007;7:85. https://bmchealthservres.biomedcentral.com/articles/10.1186/1472-6963-7-85 10.1186/1472-6963-7-85 PMC1906856 17570838

[24] Frey JH, Fontana A. The group interview in social research. Soc Sci J. 1991;28:175–87. https://www.tandfonline.com/doi/full/10.1016/0362-3319%2891%2990003-M

[25] Stratton IM. Association of glycaemia with macrovascular and microvascular complications of type 2 diabetes (UKPDS 35): prospective observational study. BMJ. 2000;321:405–12. http://www.bmj.com/cgi/doi/10.1136/bmj.321.7258.405 10.1136/bmj.321.7258.405 PMC27454 10938048

[26] Cavero-Redondo I, Peleteiro B, Álvarez-Bueno C, Rodriguez-Artalejo F.Martínez-Vizcaíno V, Glycated haemoglobin A1c as a risk factor of cardiovascular outcomes and all-cause mortality in diabetic and non-diabetic populations: a systematic review and meta-analysis. BMJ Open. 2017;7:e015949. http://www.ncbi.nlm.nih.gov/pubmed/28760792 10.1136/bmjopen-2017-015949 PMC5642750 28760792

[27] Kang CD, Tsang PPM, Li WTL, Wang HHX, Liu KQL, Griffiths SM (2015). Determinants of medication adherence and blood pressure control among hypertensive patients in Hong Kong: a cross-sectional study. Int J Cardiol.

[28] Wilson Van Voorhis CR.Morgan BL, Understanding power and rules of thumb for determining sample sizes. Tutor Quant Methods Psychol. 2007;3:43–50. http://www.tqmp.org/RegularArticles/vol03-2/p043

[29] Elm E, von, Altman DG, Egger M, Pocock SJ, Gøtzsche PC, Vandenbroucke JP. Strengthening the reporting of observational studies in epidemiology (STROBE) statement: guidelines for reporting observational studies. BMJ. 2007;335:806–8. http://www.bmj.com/lookup/doi/10.1136/bmj.39335.541782.AD 10.1136/bmj.39335.541782.AD PMC2034723 17947786

[30] Tong A, Sainsbury P, Craig J. Consolidated criteria for reporting qualitative research (COREQ): a 32-item checklist for interviews and focus groups. Int J Qual Heal Care. 2007;19:349–57. https://academic.oup.com/intqhc/article-lookup/doi/10.1093/intqhc/mzm042 10.1093/intqhc/mzm042 17872937

[31] Vaismoradi M, Turunen H, Bondas T Content analysis and thematic analysis: Implications for conducting a qualitative descriptive study. Nurs Health Sci. 2013;15:398–405. http://doi.wiley.com/10.1111/nhs.12048 10.1111/nhs.12048 23480423

[32] Braun V.Clarke V, Using thematic analysis in psychology. Qual Res Psychol. 2006;3:77–101. http://www.tandfonline.com/doi/abs/10.1191/1478088706qp063oa

[33] Patton M. Qualitative evaluation and research methods. London: SAGE Publications; 1988.

[34] Coreil J (1994). Group interview methods in community health research. Med Anthropol.

[35] Fisher K, Tenforde M, Feldstein L, Al E (2020). Community and close contact exposures associated with COVID-19 among symptomatic adults ≥18 years in 11 outpatient health care facilities—United States, July 2020. Morb Mortal Wkly Rep.

[36] Lachat C, Nago E, Verstraeten R, Roberfroid D, Van Camp J, Kolsteren P. Eating out of home and its association with dietary intake: a systematic review of the evidence. Obes Rev. 2012;13:329–46. http://doi.wiley.com/10.1111/j.1467-789X.2011.00953.x 10.1111/j.1467-789X.2011.00953.x 22106948

[37] Krishnan S, Coogan PF, Boggs DA, Rosenberg L, Palmer JR. Consumption of restaurant foods and incidence of type 2 diabetes in African American women. Am Clin Nutr. 2010;91:465–71. https://academic.oup.com/ajcn/article/91/2/465/4597226 10.3945/ajcn.2009.28682 PMC2806896 20016014

[38] Pereira MA, Kartashov AI, Ebbeling CB, Van Horn L, Slattery ML, Jacobs DR.et al. Fast-food habits, weight gain, and insulin resistance (the CARDIA study): 15-year prospective analysis. Lancet. 2005;365:36–42. https://linkinghub.elsevier.com/retrieve/pii/S0140673604176630 10.1016/S0140-6736(04)17663-0 15639678

[39] Zong G, Eisenberg DM, Hu FB, Sun Q. Consumption of meals prepared at home and risk of type 2 diabetes: an analysis of two prospective cohort studies. PLoS Med. 2016;13:e1002052. https://dx.plos.org/10.1371/journal.pmed.1002052 10.1371/journal.pmed.1002052 PMC4933392 27379673

[40] Department of Health. Report of Population Health Survey 2014/15. 2017. http://www.chp.gov.hk

[41] Littlewood JA, Lourenço S, Iversen CL, Hansen GL. Menu labelling is effective in reducing energy ordered and consumed: a systematic review and meta-analysis of recent studies. Public Health Nutr. 2016;19:2106–21. https://www.cambridge.org/core/product/identifier/S1368980015003468/type/journal_article 10.1017/S1368980015003468 PMC10270829 26714776

[42] Valdivia Espino JN, Guerrero N, Rhoads N, Simon N-J, Escaron AL, Meinen A.et al. Community-based restaurant interventions to promote healthy eating: a systematic review. Prev Chronic Dis. 2015;12:140455. http://www.cdc.gov/pcd/issues/2015/14_0455.htm 10.5888/pcd12.140455 PMC4454412 25996986

[43] Walker RJ, Gebregziabher M, Martin-Harris B, Egede LE. Quantifying direct effects of social determinants of health on glycemic control inadults with type 2 diabetes. Diabetes Technol. Ther. 2015;17:80–7. http://www.liebertpub.com/doi/10.1089/dia.2014.0166 10.1089/dia.2014.0166 PMC4322090 25361382

[44] Houle J, Lauzier-Jobin F, Beaulieu M-D, Meunier S, Coulombe S, Côté J.et al. Socioeconomic status and glycemic control in adult patients with type 2 diabetes: a mediation analysis. BMJ Open Diabetes Res Care. 2016;4:e000184. http://drc.bmj.com/lookup/doi/10.1136/bmjdrc-2015-000184 10.1136/bmjdrc-2015-000184 PMC4873951 27239316

[45] Wolfson JA, Bleich SN. Is cooking at home associated with better diet quality or weight-loss intention?. Public Health Nutr. 2015;18:1397–406. https://www.cambridge.org/core/product/identifier/S1368980014001943/type/journal_article 10.1017/S1368980014001943 PMC8728746 25399031

[46] Gallagher EJ, Le Roith D, Bloomgarden Z. Review of hemoglobin A1c in the managemen t of diabetes. J Diabetes. 2009;1:9–17. http://doi.wiley.com/10.1111/j.1753-0407.2009.00009.x 10.1111/j.1753-0407.2009.00009.x 20923515

