# Supplementary material for: Consumption of home-prepared meal at workplace as a predictor of glycated haemoglobin among people with type 2 diabetes in Hong Kong: a mixed-methods study
Source: Nutr Diabetes. 2022 Apr 4;12:16. doi: 10.1038/s41387-022-00188-1 (PMC8979147; doi:10.1038/s41387-022-00188-1)
Supplement: Supplementary file 1 — Supplementary Materials [file 41387_2022_188_MOESM1_ESM.docx]

**Appendix 1**

**Purposive sampling for focus group interviews**

Details of the heterogeneous purposive sampling strategy for the focus group interviews in relation to patients’ characteristics are explained below, with reference to the diabetes population and general population in Hong Kong.

1. **Age**: while there is no statutory retirement age in Hong Kong, we made reference to the retirement age for civil servants of Hong Kong and looked for patients not older than 65. Given the increasing trend in young onset of type 2 diabetes, we intended to include some patients in the range of 20-45 years old, while focusing on those between 45 to 65 which represents the majority of diabetes patients in our target age group.^[[1]](#footnote-1)^
2. **Sex**: Given that the prevalence of diabetes was 6.4% for female and 10.5% for male (including a higher proportion of undiagnosed diabetes for male),^1^ we intended to have at least 50% of the participants as male.
3. **Occupation**: As we would like to understand the glycaemic control of working patients with different working conditions, we intended to cover a wide range of occupations, with reference to the occupation groups among the general population in Hong Kong (there is no publicly available data on the occupations of diabetes population in Hong Kong): 44% for highly skilled occupation, 42% for medium-skilled occupation and 14% for non-skilled occupation^[[2]](#footnote-2)^.
4. **Diabetes regimen**: About 9% of diabetes patients reported that they were taking insulin.^1^ Given the more complicated regimen and self-care activities that insulin-treated patients are facing, we intended to have at least 30% of our participants to be on insulin, for a thorough understanding of their situation.

**Appendix 2**

**STROBE Statement—**

**Checklist of items that should be included in reports of cross-sectional studies**

| **Section/Topic** | **Item No** | **Recommendation** | **Reported on Page No** |
| --- | --- | --- | --- |
| **Title and abstract** | 1 | (*a*) Indicate the study’s design with a commonly used term in the title or the abstract | 1 |
|  |  | (*b*) Provide in the abstract an informative and balanced summary of what was done and what was found | 1-2 |
| **Introduction** | | | |
| Background/rationale | 2 | Explain the scientific background and rationale for the investigation being reported | 2-3 |
| Objectives | 3 | State specific objectives, including any prespecified hypotheses | 3 |
| **Methods** | | | |
| Study design | 4 | Present key elements of study design early in the paper | 4-5 |
| Setting | 5 | Describe the setting, locations, and relevant dates, including periods of recruitment, exposure, follow-up, and data collection | 6 |
| Participants | 6 | Give the eligibility criteria, and the sources and methods of selection of participants | 4&6 |
| Variables | 7 | Clearly define all outcomes, exposures, predictors, potential confounders, and effect modifiers. | 6-7 |
| Data sources/measurement | 8 | For each variable of interest, give sources of data and details of methods of assessment (measurement). Describe comparability of assessment methods if there is more than one group | 8 |
| Bias | 9 | Describe any efforts to address potential sources of bias | 6 |
| Study size | 10 | Explain how the study size was arrived at | 8 |
| Quantitative variables | 11 | Explain how quantitative variables were handled in the analyses. If applicable, describe which groupings were chosen and why | 8 |
| Statistical methods | 12 | (*a*) Describe all statistical methods, including those used to control for confounding | 8 |
|  |  | (*b*) Describe any methods used to examine subgroups and interactions | N/A |
|  |  | (*c*) Explain how missing data were addressed | 8 |
|  |  | (*d*) If applicable, describe analytical methods taking account of sampling strategy | 8 |
|  |  | (*e*) Describe any sensitivity analyses | 8 |

| **Section/Topic** | **Item No** | **Recommendation** | **Reported on Page No** |
| --- | --- | --- | --- |
| **Results** | | | |
| Participants | 13 | (a) Report numbers of individuals at each stage of study—eg numbers potentially eligible, examined for eligibility, confirmed eligible, included in the study, completing follow-up, and analysed | 12, Appendix 3 |
|  |  | (b) Give reasons for non-participation at each stage | Appendix 3 |
|  |  | (c) Consider use of a flow diagram | Appendix 3 |
| Descriptive data | 14 | (a) Give characteristics of study participants (eg demographic, clinical, social) and information on exposures and potential confounders | 12,  Table 3 |
|  |  | (b) Indicate number of participants with missing data for each variable of interest | Table 3, Table 4 |
| Outcome data | 15 | Report numbers of outcome events or summary measures | 8 |
| Main results | 16 | (*a*) Give unadjusted estimates and, if applicable, confounder-adjusted estimates and their precision (eg, 95% confidence interval). Make clear which confounders were adjusted for and why they were included | 13-14 |
|  |  | (*b*) Report category boundaries when continuous variables were categorized | N/A |
|  |  | (*c*) If relevant, consider translating estimates of relative risk into absolute risk for a meaningful time period | N/A |
| Other analyses | 17 | Report other analyses done—eg analyses of subgroups and interactions, and sensitivity analyses | 14, Appendix 5 |
| **Discussion** | | | |
| Key results | 18 | Summarise key results with reference to study objectives | 14 |
| Limitations | 19 | Discuss limitations of the study, taking into account sources of potential bias or imprecision. Discuss both direction and magnitude of any potential bias | 16-17 |
| Interpretation | 20 | Give a cautious overall interpretation of results considering objectives, limitations, multiplicity of analyses, results from similar studies, and other relevant evidence | 15-16 |
| Generalisability | 21 | Discuss the generalisability (external validity) of the study results | 16 |
| **Other Information** | | | |
| Funding | 22 | Give the source of funding and the role of the funders for the present study and, if applicable, for the original study on which the present article is based | 17 |

**Consolidated criteria for reporting qualitative studies (COREQ): 32-item checklist**

| **No. Item** | **Guide questions/description** | **Reported on Page #** |
| --- | --- | --- |
| **Domain 1: Research team and reﬂexivity** | | |
| *Personal Characteristics* |  |  |
| 1. Inter viewer/facilitator | Which author/s conducted the interview or focus group? | 5 |
| 2. Credentials | What were the researcher’s credentials? E.g. PhD, MD | 1 |
| 3. Occupation | What was their occupation at the time of the study? | N/A |
| 4. Gender | Was the researcher male or female? | N/A |
| 5. Experience and training | What experience or training did the researcher have? | 1 |
| *Relationship with participants* |  |  |
| 6. Relationship established | Was a relationship established prior to study commencement? | 5 |
| 7. Participant knowledge of the interviewer | What did the participants know about the researcher? e.g. personal goals, reasons for doing the research | 5 |
| 8. Interviewer characteristics | What characteristics were reported about the inter viewer/facilitator? e.g. Bias, assumptions, reasons and interests in the research topic | N/A |
| **Domain 2: study design** |  |  |
| *Theoretical framework* |  |  |
| 9. Methodological orientation and Theory | What methodological orientation was stated to underpin the study? e.g. grounded theory, discourse analysis, ethnography, phenomenology, content analysis | 7 |
| *Participant selection* |  |  |
| 10. Sampling | How were participants selected? e.g. purposive, convenience, consecutive, snowball | 4,  Appendix 1 |
| 11. Method of approach | How were participants approached? e.g. face-to-face, telephone, mail, email | 4 |
| 12. Sample size | How many participants were in the study? | 9 |
| 13. Non-participation | How many people refused to participate or dropped out? Reasons? | 9 |
| *Setting* |  |  |
| 14. Setting of data collection | Where was the data collected? e.g. home, clinic, workplace | 4 |
| 15. Presence of non-participants | Was anyone else present besides the participants and researchers? | 5 |
| 16. Description of sample | What are the important characteristics of the sample? e.g. demographic data, date | 9,  Table 1 |
| *Data collection* |  |  |
| 17. Interview guide | Were questions, prompts, guides provided by the authors? Was it pilot tested? | 5 |
| 18. Repeat interviews | Were repeat inter views carried out? If yes, how many? | N/A |
| 19. Audio/visual recording | Did the research use audio or visual recording to collect the data? | 5 |
| 20. Field notes | Were ﬁeld notes made during and/or after the interview or focus group? | 5 |
| 21. Duration | What was the duration of the interviews or focus group? | 5 |
| 22. Data saturation | Was data saturation discussed? | 5 |
| 23. Transcripts returned | Were transcripts returned to participants for comment and/or correction? | N/A |
| **Domain 3: analysis and ﬁndings** | | |
| *Data analysis* |  |  |
| 24. Number of data coders | How many data coders coded the data? | N/A |
| 25. Description of the coding tree | Did authors provide a description of the coding tree? | Figure 2 |
| 26. Derivation of themes | Were themes identiﬁed in advance or derived from the data? | 7-8 |
| 27. Software | What software, if applicable, was used to manage the data? | 8 |
| 28. Participant checking | Did participants provide feedback on the ﬁndings? | N/A |
| *Reporting* |  |  |
| 29. Quotations presented | Were participant quotations presented to illustrate the themes/ﬁndings? Was each quotation identiﬁed? e.g. participant number | Table 2 |
| 30. Data and ﬁndings consistent | Was there consistency between the data presented and the ﬁndings? | 11 |
| 31. Clarity of major themes | Were major themes clearly presented in the ﬁndings? | 10-11,  Table 2 |
| 32. Clarity of minor themes | Is there a description of diverse cases or discussion of minor themes? | 10-11,  Table 2 |

**Appendix 3**

Type 2 diabetes patients randomly selected and approached: n=422

Patients refused to participate: n=90

Patients agreed to participate:

n=332 (response rate: 78.7%)

Type 2 diabetes patients included in the cross-sectional study:

n=185

Incomplete questionnaires:

n=21

Ineligible patients

(without employment):

n=126

Questionnaires filled out by patients with employment:

n=206

**Supplementary Figure 1.** Number of samples of the cross-sectional study

**Appendix 4**

**Assumptions testing of cross-sectional data for multiple linear regression**

The cross-sectional data analysed in this study met the various assumptions for multiple linear regression. Details as follows –

1. There was approximate linearity between the dependent variable of HbA1c and individual continuous independent variables, as assessed by partial regression plots, and all independent variables collectively, shown in a plot of studentized residuals against the predicted values.
2. Given the study design, it was highly unlikely for the observations to be related.
3. There was homoscedasticity, as assessed by visual inspection of a plot of studentized residuals versus unstandardized predicted values.
4. There was no evidence of multicollinearity, as tolerance values for all independent variables were greater than 0.1.
5. The assumption of normality of residuals was met, as assessed by Q-Q Plot.
6. There were no leverage values greater than 0.2, and no values for Cook's distance above 1. The three potential outliers (defined as those with studentized deleted residuals greater than ±3 standard deviations) were not removed from the main analysis since they were neither high leverage points nor influential points, and it was unlikely that the outliers existed due to input error as they were retrieved directly from electronic records of the clinic.

**Appendix 5**

**Supplementary Table 1:** Comparing results of primary analysis and sensitivity analyses (Model 4 only)

|  |  |  | HbA1c (%) | |  |  |
| --- | --- | --- | --- | --- | --- | --- |
|  | Primary analysis ^a^ | | Sensitivity analysis 1 ^b^ | | Sensitivity analysis 2 ^c^ | |
| Variable | B | β | B | β | B | β |
| Constant | 7.187* |  | 6.942* |  | 7.365* |  |
| Age | .026 | .013 | .075 | .038 | .080 | .046 |
| Sex | -.006 | -.039 | -.003 | -.024 | -.013 | -.100 |
| Education level (primary) | -.132 | -.057 | -.161 | -.070 | -.056 | -.027 |
| Education level (secondary) | -.288 | -.157 | -.293 | -.159 | -.163 | -.102 |
| Occupation (non-skilled) | -.105 | -.050 | -.105 | -.050 | -.192 | -.104 |
| Occupation (medium-skilled) | -.286 | -.163 | -.257 | -.146 | -.314 | -.206 |
| Monthly income (HK$0-9999) | .281 | .140 | .273 | .134 | .377 | .215 |
| Monthly income (HK$10000-29999) | .337 | .189 | .330 | .183 | .466* | .300 |
| Current smoker | -.004 | -.002 | .018 | .008 | -.042 | -.024 |
| Exercise outside home and work | -.107 | -.061 | -.076 | -.043 | -.160 | -.105 |
| Diabetes duration | .035* | .226 | .041* | .250 | .042** | .314 |
| Presence of comorbidities | .103 | .049 | .099 | .047 | .068 | .037 |
| Consumption of HPM (regular) | -.475* | -.221 | -.509* | -.235 | -.401* | -.216 |
| Consumption of HPM (occasional) | .002 | .001 | -.008 | -.003 | -.095 | -.039 |
|  |  |  |  |  |  |  |
| R^2^ | .146 |  | .155 |  | .211 |  |
| Δ R^2^ | .044* |  | .048* |  | .039* |  |

Note.

* p < 0.05, ** p <0.001

a = Primary analysis covered all subjects (n=185)

b = Sensitivity analysis 1 excluded insulin-treated patients (n=181)

c = Sensitivity analysis 2 excluded outliers in HbA1c (%) (n = 182); outliers defined as those with studentized deleted residuals greater than ±3 standard deviations

1. Department of Health. Report of Population Health Survey 2014/15. Available from: http://www.chp.gov.hk [↑](#footnote-ref-1)
2. Number of employed persons by industry and occupation (2018), Census and Statistics Department, Hong Kong Government. Available from https://www.censtatd.gov.hk/hkstat/sub/sp200.jsp?productCode=D5250003 [↑](#footnote-ref-2)
